# Studies of Reduced Graphene Oxide and Graphite Oxide in the Aspect of Their Possible Application in Gas Sensors

**DOI:** 10.3390/s16010103

**Published:** 2016-01-15

**Authors:** Sabina Drewniak, Roksana Muzyka, Agnieszka Stolarczyk, Tadeusz Pustelny, Michalina Kotyczka-Morańska, Maciej Setkiewicz

**Affiliations:** 1 Department of Optoelectronics, Silesian University of Technology, 2 Akademicka Str., Gliwice 44-100, Poland; Tadeusz.Pustelny@polsl.pl (T.P.); Maciej.Setkiewicz@polsl.pl (M.S.); 2 Institute for Chemical Processing of Coal, 1 Zamkowa Str., Zabrze 41-803, Poland; rmuzyka@ichpw.pl (R.M.); mmoranska@ichpw.pl (M.K.-M.); 3 Department of Physical Chemistry and Technology of Polymers, Silesian University of Technology, 9 Strzody Str., Gliwice 44-100, Poland; Agnieszka.Stolarczyk@polsl.pl

**Keywords:** graphite oxide, graphene oxide, gas sensor, explosive detection

## Abstract

The paper presents the results of investigations on resistance structures based on graphite oxide (GRO) and graphene oxide (rGO). The subject matter of the investigations was thaw the sensitivity of the tested structures was affected by hydrogen, nitrogen dioxide and carbon dioxide. The experiments were performed at a temperature range from 30 °C to 150 °C in two carrier gases: nitrogen and synthetic air. The measurements were also aimed at characterization of the graphite oxide and graphene oxide. In our measurements we used (among others) techniques such as: Atomic Force Microscopy (AFM); Scanning Electron Microscopy (SEM); Raman Spectroscopy (RS); Fourier Transform Infrared Spectroscopy (FT-IR) and X-ray Photoelectron Microscopy (XPS). The data resulting from the characterizations of graphite oxide and graphene oxide have made it possible to interpret the obtained results from the point of view of physicochemical changes occurring in these structures.

## 1. Introduction

The development of industry, agriculture, medicine and public safety requires the concomitant development of suitable control and monitoring systems [1]. This is connected with the need to provide selective and sensitive sensors [2] as the worldwide progress of civilization and technology demands better and better sensor solutions. This can be achieved by modification of already existing solutions or finding new ones, which involve the necessity of investigating the modification of already existing possibilities and endeavoring to find new ones which might detect changes in the natural work environment. Based on research conducted all over the world, it can be stated that materials like ZnO [3], TiO_2_ [4] and WO_3_ [5] can serve as excellent sensing layers. Also hybrid materials of conductive polymers with two-dimensional nanofilters ought to be taken into account (e.g., graphene oxide-poly (3-hexylthiophene) nanocomposites) [6]. It is to be stressed that the use of carbon materials just for decorating for instance with palladium [7,8] or for use in hybrids (and also in pristine form) are becoming more and more interesting, not only in the sensor domain The range of potential applications of carbon structures is rather wide and information provided in the respective literature indicates that carbon nanomaterials may be potentially used, among others, as selective molecular membranes [9]. The applications of graphene, graphene oxide and reduced graphene oxide in the production of sensors is also a very promising field [10,11].

The techniques applied for the preparation of graphite oxide [12] and its further reduction [13] to reduced graphene oxide affect the properties of these materials, due to the presence of additional functional groups. Graphite oxide contains, among others, many hydroxyl, carboxyl [14] and epoxy [15] groups. The amount of these groups on graphene sheets directly influences the electric properties of the material. An increase in the amount of functional groups reduces the electric conductivity, which in turn would result in an increase in the electric mobility [13,15]. Thus, graphite oxide and graphene oxide can be used in a resistance sensor making it possible to detect selected gaseous atmospheres.

In this paper, the authors present the results of experiments aimed at investigating the responses of resistance structures based on graphite oxide and graphene oxide exposed to different atmospheres. We also present the results of characterization of the layers comprising, among others, an analysis of the composition and topography of the surfaces. The aim of our investigations was to understand the physicochemical phenomena occurring during contact of the sensor structures with the given gaseous atmospheres.

Our investigations were focused on the detection of three dangerous gases: nitrogen dioxide, hydrogen and carbon dioxide. Gases from the NO_x_ group pollute the natural environment, leading to the formation of smog and affecting the lungs [16]. Hydrogen is harmful because it may already cause explosions at a concentration of 4% in air [17]. Carbon dioxide belongs to the so-called thermal gases (greenhouse gases) [18]. Both hydrogen and carbon dioxide are colorless, non-aromatic and tasteless gases [13,17] and that is why they should be detected.

## 2. Experimental Sction

### 2.1. Preparation of Graphite Oxide and Graphene Oxide

Commercial natural graphite powder (90 µm, L_a_-58 nm, L_c_-30 nm, d_002_-0.336 nm and C^daf^-99.5%), supplied by Graphit Kropfmühl AG (Hauzenberg, Germany) was ground and sieved to a particle size <20 µm and oxidized according to the methodology described in Table 1.

**Table 1:** Reaction conditions for the preparation of GRO.

Sample	Reactants	Reaction Time	Reference
Graphite oxide	Graphite (1 g); H_2_SO_4_ (30 mL); NaNO_3_ (3 g); KMnO_4_ (3 g)	2 h	[19]

**Figure 1:**
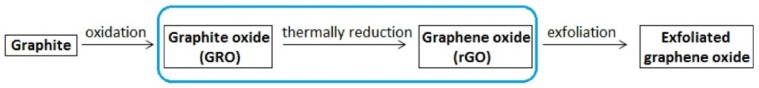
Preparation of graphite oxide and graphene oxide.

Concentrated H_2_SO_4_ (95%–97%) was used as an acid. KMnO_4_ and NaNO_3_ were slowly added to keep the temperature in the 10–15 °C range. After their addition, the reaction temperature was maintained between 10 and 20 °C. After each reaction, 120 mL of milli-Q water and 50 mL of 3% H_2_O_2_ were slowly added. The mixture was stirred for 30 min and then centrifuged, the supernatant being decanted off. The graphite oxide was washed with milli-Q water and centrifuged repeatedly until a neutral pH was achieved. Graphite oxide was finally dried overnight under vacuum at 50 °C and stored in the presence of P_2_O_5_ as desiccant. Graphene oxide was obtained from graphite oxide by thermal treatment at 900 °C in a vertical furnace, under a nitrogen flow of 50 mL/min. The residence time at the final temperature was 5 min. Figure 1 shows the preparation of graphite oxide and graphene oxide.

### 2.2. Applied Measurement Methods

Both graphite oxide (GRO) and graphene oxide (rGO) possess unique properties that differ from those of pristine graphite because of the structural changes arising due to the introduction of oxygen functionalities into the *sp^2^* bonded carbon network. We used a combination of several measurement methods to reveal the structural evolution from pristine graphite to graphite oxide and next to graphene oxide (upon thermally reduction and exfoliation). The further sections discuss the results obtained using these methods.

The topography of the surface of graphite oxide and graphene oxide was investigated by means of Scanning Electron Microscopy (SEM) and Atomic Force Microscopy (AFM). For measurements using Scanning Electron Microscopy, an INSPECT S50 (FEI, Hillsboro, Oregon, OH, USA) instrument was used. The measurement parameters were: HV = 5 kV, bias = 0, spot = 3.0 and HV = 2 kV, bias = 1400 V, spot = 3.5 for graphite oxide and graphene oxide, respectively. In order to illustrate both materials, an Everhart-Thornley Detector (ETD) was used. The measurements were made under high vacuum conditions (1.19 × 10^−5^ mbar). The microscope magnification was ×1000 and ×5000. Additionally, the investigations by means of scanning electron microscopy were extended by applying an energy dispersive spectrometer X-ray (EDX) detector. This detector allows for qualitative and quantitative analysis of the sample composition. The measurements were examined by using a Nova NanoSEM 450 microscope (FEI, Hillsboro, Oregon, OH, USA) operating under low vacuum (0.3 mbar) at an accelerating voltage of 10–15 kV and using a large-field secondary electron detector.

The results obtained by applying SEM microscopy were supplemented by the results from AFM microscopy. Investigations using this technique were performed on an N_TEGRA Prima platform (NT-MDT, Moscow, Russia) using the intermittent contact mode. The images were obtained at a resonance frequency equal to 136.281 kHz, both for graphite oxide and graphene oxide. The HA_NC tip was used in those measurements. Moreover, the Raman Spectroscopy (RS), Fourier Transform Infrared Spectroscopy (FT-IR), X-ray Diffraction (XRD) and X-ray Photoelectron Microscopy (XPS) were also applied for the characterization of the layers. The Raman spectra were obtained from the N_TEGRA Spectra platform (NT-MDT). In the measurements, the wavelength was equal to 532 nm. The spectra were recorded with a resolution of 2.89 cm^−1^.

FT-IR measurements were made with a Tensor 27 spectrometer (Bruker, Ettlingen, Germany). Absorbance spectra of discs with a KBr/sample ratio of 500:1 were collected at a resolution of 4 cm^−1^.

XRD measurements were taken with an X’Pert PRO PW 3040/60 by PANalytical B.V. (Quebec, QC, Canada) diffractometer. Samples of ground graphite, graphite oxide and thermally reduced graphene oxide were deposited onto glass and analyzed by using Co Ka1 radiation with a voltage of 40 kV and a current of 30 mA.

The elements present in graphite oxide and graphene oxide as well as their chemical state were identified by XPS analysis. X-ray Photoelectron Microscopy was carried out using a PHI 5000 VersaProbe—Scanning ESCA Microprobe™ (ULVAC-PHI, Chigasaki, Japan/Chanhassen, MN, USA) and a monochrome Al Kα source (1486.6 eV). The charging of the sample was corrected applying the C1s peak at 284.5 eV as an internal standard. Curve fitting of the spectra was performed using a Gaussian-Lorentzian peak shape after conducting a Shirley background correction. The high-resolution C1s signal was deconvoluted into five individual peaks ascribed to graphitic carbon (284.5 eV), carbon atoms with *sp^3^* hybridization (285.4 eV), hydroxyl and epoxy groups (286.5 eV), carbonyl or quinone groups (287.6 eV), carboxyl groups (288.9 eV) and a satellite peak corresponding to the π–π* transition in the aromatic systems (290.4 eV). The O1s excitation was resolved into two peaks corresponding to C=O groups such as ketone and carbonyl (531.7 eV), hydroxyl and epoxy functionalities (533.4 eV) and a carboxyl group (535.3 eV). Curve fittings were performed using an iterative least squares algorithm (CasaXPS software) with a Gaussian-Lorentzian (70/30) peak shape and Shirley background removal. The resulting spectra represent the binding energies of pyridinic (398.5 ± 0.2 eV), amine (399.4 ± 0.2 eV), hydroxypyridinic (400.5 ± 0.2 eV), quaternary-N (401.2 ± 0.2 eV) and N-oxide (402.9 ± 0.2 eV) groups.

The elementary composition of graphite oxide and graphene oxide (carbon, oxygen, hydrogen, nitrogen and sulphur content) was determined directly using a Vario Macro Cube automatic elementary analyser (Elementar Analysysteme GmbH Company, Hanau, Germany).

The properties of graphite oxide and graphene oxide as a function of temperature were investigated by the following techniques: Thermogravimetric (TG), Differential Thermogravimetric (DTG) and Differential Scanning Calorimetry (DSC). TG, DTG and DSC curves were obtained using a TG-STA209LUXX unit (Netzsch Group, Selb, Germany). The samples were tested in the range from 40 to 1000 °C with a heating rate of 10 K/min and a flow of argon kept at 25 mL/min.

The resistance of sensor samples as a function of temperature was tested in a measuring system designed and realized by the staff of the Department of Optoelectronics of the Silesian University of Technology (Gliwice, Poland). This system allows presetting and control of the temperature of samples. The measurements were made within the range of temperature from 30 °C to 150 °C. The same system made it possible to measure the resistance at various degrees of temperature in various gaseous atmospheres. It can preset and control the composition of the gaseous atmosphere over the samples (the gas flow: 100 mL/min and 500 mL/min in the undertaken experiments). The measurements were taken in the atmospheres of nitrogen and synthetic air, which were used as carrier gases, as well as in the atmosphere of hydrogen, nitrogen dioxide and carbon dioxide at various concentrations in the carrier gases.

### 2.3. Description of a Sensor Structure

**Figure 2:**
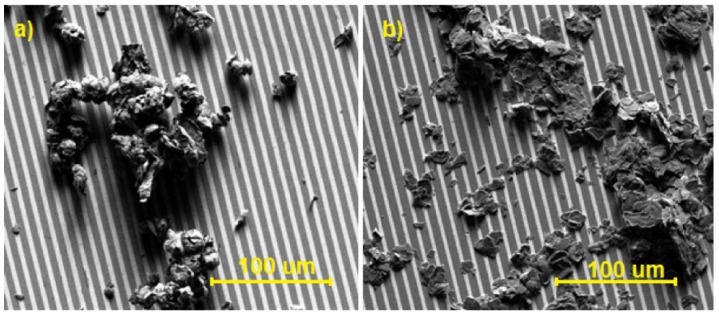
Photos of sensor structures obtained using Scanning Electron Microscopy (magnification: ×1000): (**a**) structure with graphite oxide; (**b**) structure with graphene oxide.

The tested sensor structures (Figure 2) differed from each other merely in the sensitivity of the layers–the sensitive layers were deposited on the same substrates (the so-called “basic substrates”). In order to get basic substrates, gold comb electrodes were deposited on silicon substrates (with an oxidized surface). A layer of chromium was sputtered to improve the adhesion of gold to the substrate.

To obtain a 1.2% (wt.%) solution the appropriate amounts of graphite oxide and graphene oxide (sensitive layers) were mixed with ethanol (ethyl alcohol absolut 99.8% pure, Avantor Performance Materials S.A, Gliwice, Poland) and then transferred to an ultrasonic bath (Transsonic T310, Clamlab Ltd., Cambridge, UK). After 24 h ultrasonication a homogeneous dispersion was obtained, which was deposited on the basic structures. After that the basic structures were placed in an vacuum dryer (Memmert VO400, Schwabach, Germany) and dried at 22 °C under nitrogen atmosphere for 24 h. Next, the dried structures were annealed with temperature ramp 2 °C/min to the final temperature of 100 °C at which were maintained for additional 3 h. Afterwards the temperature was increased up to 150 °C using the same ramping program and kept for 2 h under reduced pressure (20 mbar) to removal of any residual ethanol from the prepared structures. The whole annealing process were conducted in an inert atmosphere of nitrogen to avoid oxidation of the samples.

The surface of the sensors structures was about 1 cm^2^. The application of the sensitive layer on such large surfaces caused that the obtained results were repetitive for different groups of sensors (the resistance of the structure with graphite oxide was approximately equal to 48 Ω, while the resistance of the structure with graphene oxide was approximately equal to 5.8 Ω.

As it is shown in SEM images (Figure 2), the thickness of the GRO and rGO is relatively large compared to many structures presented in the literature [20,21,22]. We made numerous attempts to exfoliate layers. The resulting layers were delaminated but their properties were not stable (after some time, the layers agglomerated again). We wanted to make the layers stable over time and thermally stable. The structures with such layers are presented in Figure 2.

## 3. Results and Discussion

### 3.1. Characterization of the Structures—Topography and Composition of Graphite Oxide and Graphene

**Figure 3:**
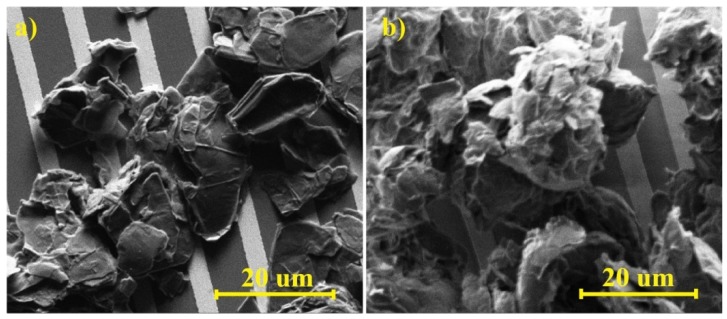
Photos of sensor structures obtained by using Scanning Electron Microscopy (magnification of ×5000): (**a**) structure with graphite oxide; (**b**) structure with graphene oxide.

Figure 3 shows the photos obtained by using Scanning Electron Microscopy (magnification of 5000 times), concerning both graphite oxide and graphene oxide. The size of the grains of both oxides is similar, from only a few to a score of micrometers, although the surface of graphite oxide is distinctly more developed. The structure of graphite oxide is smoother than that of graphene oxide; moreover, it displays a parallel arrangement of the respective layers (Figure 3a).

**Figure 4:**
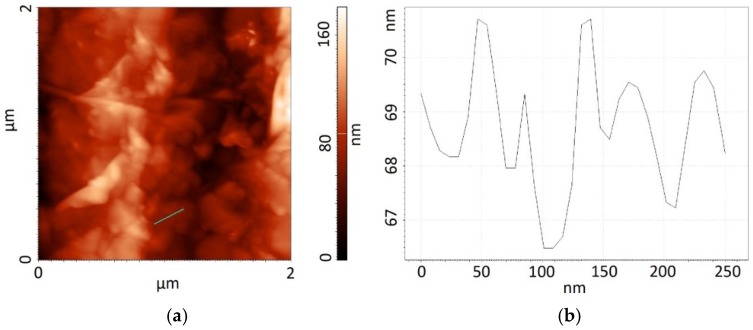
Topography of the surface of graphite oxide: (**a**) picture obtained by using AFM; (**b**) cross-sections of the marked area.

**Figure 5:**
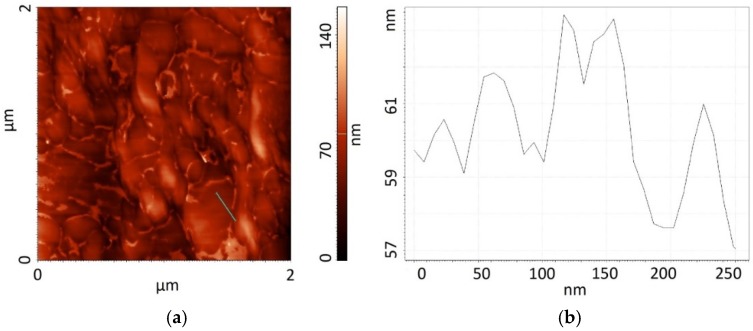
Topography of the surface of graphene oxide: (**a**) picture obtained by using AFM; (**b**) cross-sections of the marked area.

The results of tests carried out when applying Atomic Force Microscopy confirm the difference in the development of these structures. The topography (2 × 2 µm^2^) of the surface of graphene oxide is presented in Figure 4a. The topography of graphite oxide changes within a larger range (from 0 to 170 nm) than that of graphene oxide (from 0 to 150 nm). Analyzing the cross-sections (Figure 4b and Figure 5b) across the surface (marked by the blue lines in Figure 4a and Figure 5a), we see that both materials, the changes in height amount to several nanometers along the cross-section of 250 nm.

The value of the RMS coefficient for graphite oxide amounts to RMS = 15.98 nm, whereas for graphene oxide to RMS = 28.51 nm, which proves the better development of the surface of graphene oxide.

**Figure 6:**
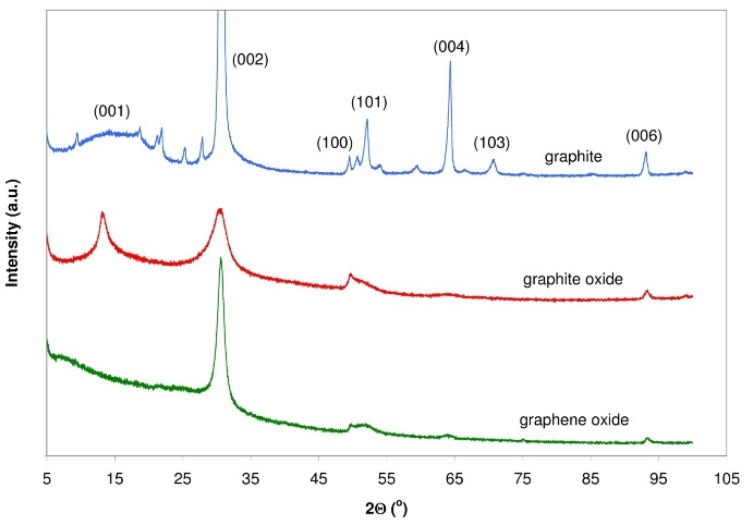
X-ray patterns for the parent graphite, GRO and rGO treated at 900 °C.

The crystalline structure of synthesized graphite oxide and graphene oxide, as well as that of the parent graphite, were analysed by XRD. As shown in Figure 6, the XRD pattern of the graphite shows a diffraction peak at 2Θ = 30.87°, corresponding to its interlayer spacing (d_002_) of 0.3363 nm. The intensity of this peak sharply decreases for GRO, and a new 001 peak appears at 2Θ = 13.18°. The d_001_ interlayer distance calculated for this sample is 0.7795 nm, which is in agreement with its high degree of oxidation [23,24]. The variation in the interlayer spacing of GRO results from the variation in the degree of oxidation on graphite and is proportional to the content of oxygen. Apart from the characteristic sharp diffraction peak at 2Θ approximately 13°, displays mildly oxidised RGO exhibits another weak peak at 2Θ = 30°. In the case of thermal reduction of GRO, the characteristic peak of GRO at 2Θ = 13.18° has been reported to disappear and a new broad peak appears at 2Θ = 30.50°. The decrease in the interlayer spacing between the thermally reduced graphene oxide sheets is attributed to the removal of considerable oxygen functionalities from the GRO sheet during the reduction process. The interlayer spacing d_002_ amounts to 0.3393 nm.

**Figure 7:**
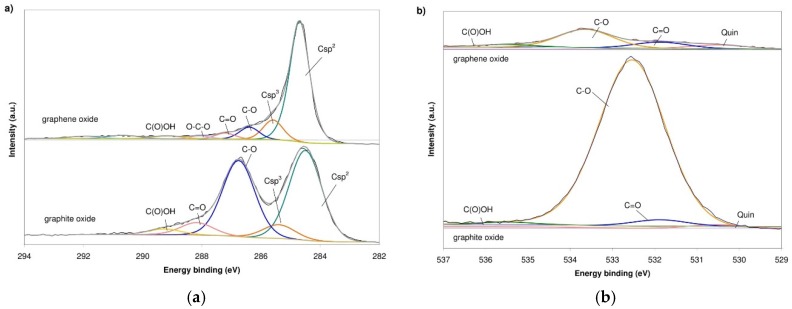
Deconvolution of (**a**) C1s and (**b**) O1s core-level XPS spectra of the GRO and rGO.

The oxygen speciation was studied by means of XPS measurements. Figure 7a shows the C1s core-level XPS spectra of graphite and graphene oxides. The spectra of GRO show an intense peak at 284.5 eV, attributed to carbon with *sp^2^* and *sp^3^* hybridization accompanied by some shoulders at higher binding energies due to the presence of oxygen linkages [25,26]. The samples also show a second intense peak at 286.5 eV, ascribed to hydroxyl and epoxy groups, and less intense contributions of carbonyl/quinone (287.6 eV) and carboxyl (288.9 eV) groups. The oxygen speciation in these samples is in agreement with the model of Lerf-Klinowski concerning highly oxidized graphitic structures [27]. The spectrum of rGO shows an intense peak at 284.5 eV attributed to carbon with *sp^2^* hybridization. The C1s spectrum consists of a peak at 285.4, 286.2, 287.0, 287.9, 288.9, and 290.4 eV assigned to C–C, C–O, C=O, O–C–O, O=C–OH and π–π* transition in aromatic systems [28].

The O1s core-level spectra for GRO, shown in Figure 7b , were deconvoluted into four peak; to quinone (530.3 eV), oxygen double-bonded to carbon (531.7 eV), oxygen single-bonded to carbon (532.5 eV) and carboxylic group (535.3 eV). The O1s core-level spectra for rGO, shown in the same diagram, were deconvoluted into four peaks, ascribed to quinone (530.3 eV), oxygen double-bonded to carbon (531.7 eV), oxygen single-bonded to carbon (533.4 eV) and carboxylic group (535.3 eV) [28,29]. The results of the deconvolution of the C1s and O1s XPS spectra are gathered in Table 2.

**Table 2:** Elemental composition of GRO and rGO determined by XPS.

Sample Code	Elemental Composition (%)		C1s Deconvolution						O1s Deconvolution			
	C	O	C*sp^2^*	C*sp^3^*	C–O	C=O	O–C–O	C(O)OH	C–O	C=O	C(O)OH	Quinone Groups
GRO	69.0	31.0	45.4	7.0	38.5	6.2	-	2.8	83.0	**9.2**	**2.7**	**5.1**
rGO	94.5	5.5	70.5	11.0	6.7	3.3	1.7	2.6	58.5	20.4	8.5	12.7

**Figure 8:**
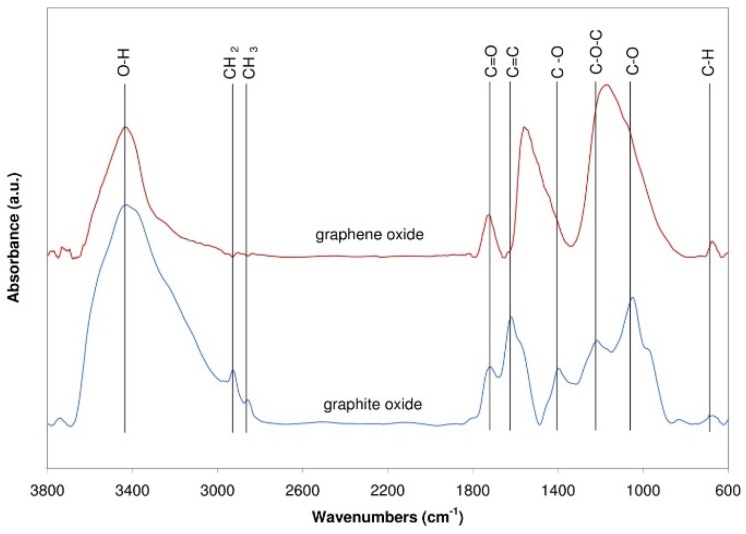
FT-IR spectra for graphite oxide and graphene oxide.

The FT-IR spectra of the samples of graphite and graphene oxides are presented in Figure 8. In all the spectra, the oxidation is confirmed by the presence of several bands attributed to oxygen functionalization. As evident in Figure 8, there are groups of oxidized graphite FT-IR absorbance peaks. The most intense peak (*i.e.*, the widest) occurs in the range of 3320–3430 cm^−1^ and is attributed to the C–OH stretching vibrations of a hydroxyl group [30,31,32,33,34]. A second, lower-intensity peak is located at approximately 1620 cm^−1^ and is attributed to the C=C skeletal vibration of non-oxidized graphite [31,33,34]. A third peak with a large intensity, comparable to that of the second peak, is located at approximately 1050 cm^−1^ and is attributed to the alkoxy C–O stretching vibration [30,31,32,34].

The next peak with the lowest intensity is located at approximately 1730 cm^−1^ and is attributed to the C=O stretching vibrations of a carbonyl group (at the edges of planes of the graphite layer) [30,31,32,34,35]. Two weak peaks located at approximately 2970 and 2940 cm^−1^ are characteristic for asymmetric and symmetric stretching vibrations of the C-H bond in CH_2_ and CH_3_ groups [31,34]. The next group of the peaks contains peaks which can be observed also in the spectra of graphene oxide. The most intense (*i.e.*, the widest) peak is located at approximately 1220 cm^−1^ and is attributed to the stretching vibration of the epoxy group C–O–C [30,31,32,34,36]. A second, lower-intensity peak is located at approximately 1560 cm^−1^ and is attributed to the C=C skeletal vibration of the graphene planes [31,33,34,36]. A third peak with reduced intensity compared to that of graphite oxides, at approximately 3440 cm^−1^, is attributed to C–OH stretching vibrations of a hydroxyl group [30,31,32,33,34,36]. The intensity of this peak is closely related to the oxygen content in the tested samples after high-temperature treatment. Finally, a fourth peak with the most reduced intensity, visible also in the spectrum of graphene oxide, (located at approximately 1710 cm^−1^) is attributed to the stretching vibration of the carbonyl group C=O [30,31,32,34,35,36].

**Figure 9:**
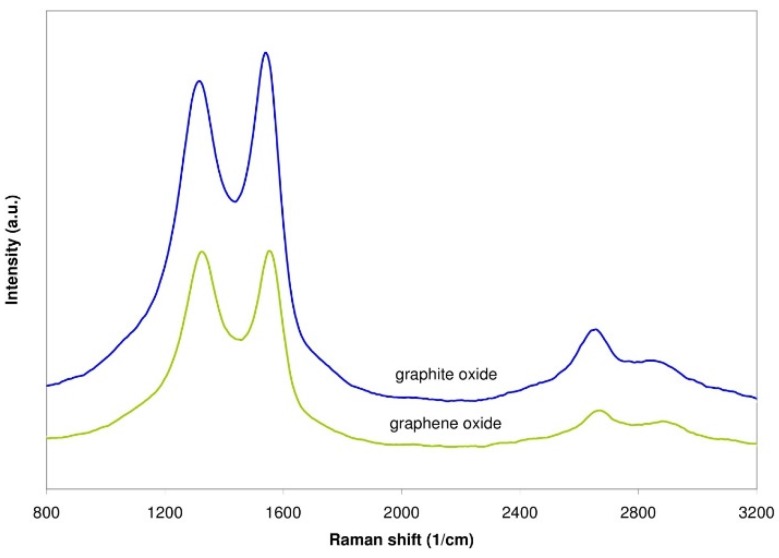
Raman spectra of graphite oxide and graphene oxide.

The Raman spectra of graphite oxide and graphene oxide are shown in Figure 9. The two most intensive peaks occur in the ranges 1300–1400 cm^−1^ (D) and 1550–1600 cm^−1^ (G) [37]. The ratio of the intensities of the D and G peaks for graphite oxide is equal to 0.90, whereas for graphene oxide it equals 0.98. The growth of this ratio suggests that the amount of defects increased.

**Figure 10:**
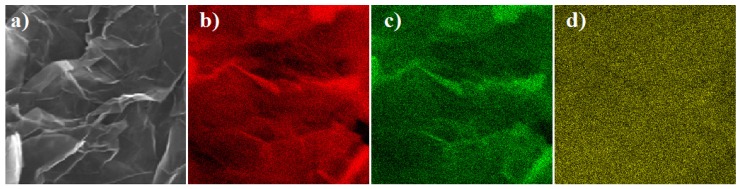
SEM image of (**a**) graphene oxide and (**b**) EDX maps of the corresponding area for carbon; (**c**) oxygen and (**d**) sulphur.

The presence of additional elements in the analyzed structures was also confirmed by application of other methods of measurements, for example Scanning Electron Microscopy with the EDX detector. Figure 10a shows highly-wrinkled graphitic layers proving that there is a distortion in the graphene layers due to the linkage of the residual oxygen after thermal reduction, while large nanosheet sizes are preserved.

EDX maps are shown in Figure 10b–d; carbon, oxygen and sulfur are detected on the surface of both samples and the presence of sulfur on the surface is a striking difference. The amount of elements detected by using various methods is summarized in Table 3. One has to be aware of the fact that both EDX and XPS detect the content of elements on the surface, while EA is a complex analysis.

**Table 3:** Elemental composition (of carbon, oxygen and sulphur) of graphite oxide and graphene oxide determined by XPS, EDX and EA methods.

	C_XPS_, %	O_XPS_, %	C_EDX_, %	O_EDX_, %	S_EDX_, %	C_EA_, %	O_EA_, %	S_EA_, %
Graphite oxide	69.0	31.0	68.9	26.70	4.40	63.80	32.29	1.62
Graphene oxide	94.5	5.5	89.5	8.50	2.00	89.80	8.53	0.95

Nevertheless, the analyses are in quite good agreement, but the EDX and EA data confirm the presence of sulphur in the samples. On the basis of the maps, it may be concluded that the samples are very uniform over the entire volume.

### 3.2. Characteristics of the Structures—The Dependence of Selected Properties on the Temperature

The properties of graphite oxide and graphene oxide change together with the temperature, affecting directly the properties of sensor structures based on the mentioned materials. Therefore, measurements were accomplished using the TG/DTG, and DSC techniques and also measuring the resistance at various degrees of temperature.

**Figure 11:**
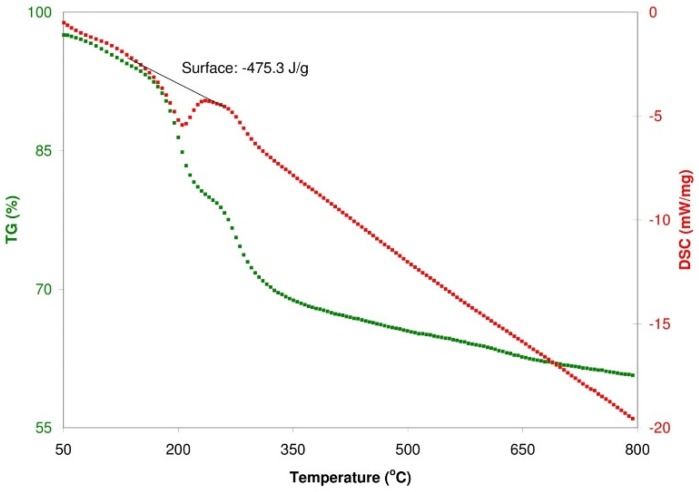
Contains onset degradation temperatures, residual material, melting points and melt enthalpy from TGA and DSC analysis for GRO.

Analysing the data from Figure 11, it can be seen that pyrolysis occurs in several steps. In the first step (from 40 °C to 140 °C), the maximum DTG temperature amounts to 100 °C (the change in the weight is then equal to 3.6%) The weight change is (in this case) associated with a loss of moisture in the samples. The second pyrolysis step occurs within the range of temperature from 140 °C to 240 °C and from 240 °C to 1000 °C. In the temperature range from 140 °C to 240 °C, the maximum DTG temperature is equal to 200 °C, connected with a loss of weight of: 14.2%, while in the temperature range from 240 °C to 1000 °C, the maximum DTG temperature amounts to 275 °C with a loss of weight of: 19.2%. The second step of pyrolysis is associated with a disconnection of the functional groups from the surface (from the oxidized graphene planes) [38]. DSC data shown in Figure 11 reveal that for GRO an exothermic peak is observed between 140 °C and 240 °C with peak location at 200 °C. This peak corresponds to the melting point of the material, specifically the melting of γ phase crystals. The melt enthalpy is −475.3 J/g [39].

Sensor structures are also characterized by various resistance values. In the course of the oxidation process, the incorporated oxygen overcomes the van der Waals bonds and as a result, the interplanar distances increase. The reduction of graphene oxide eliminates a considerable amount of oxygen and changes the impedance characteristic of the material. A reduction in the amount of groups containing oxygen increases the electronic conduction, resulting in a decrease of resistance.

**Figure 12:**
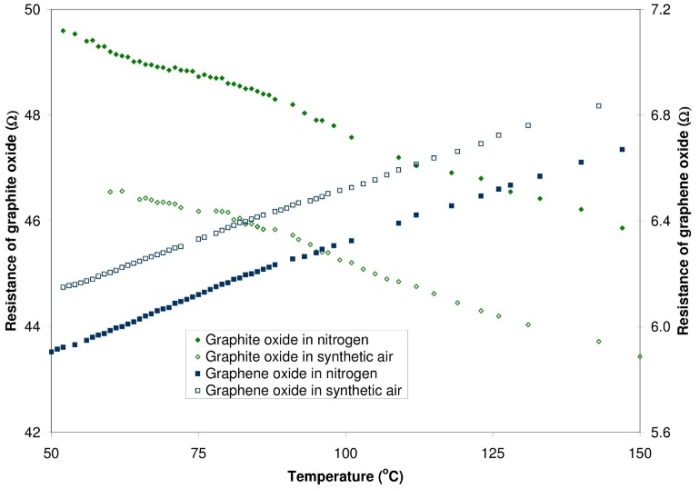
Changes of the resistance of structures with graphite oxide and graphene oxide *vs.* temperature—the measurements in nitrogen and synthetic air.

It ought to be mentioned that the resistance of graphite oxide changes in a different way with the rise of temperature than the resistance of graphene oxide. The conductivity of a structure containing graphite oxide increases with the rise of temperature (within the range from 50 °C to 150 °C), both in nitrogen and in synthetic air. The character of these changes is linear. In the case of nitrogen, the rate of changes amounts to 37.7 mΩ/°C, whereas for synthetic air it amounts to 38.6 mΩ/°C. In the structure with graphene oxide, the resistance grows with the increasing temperature. For nitrogen, the rate of changes amounts to 7.8 mΩ/°C while for synthetic air it is equal 8.1 mΩ/°C. The results are presented in Figure 12.

The different conductivity characteristics are probably due to the changes occurring in the atomic bonds of materials. The connection of oxygen to graphite (during the formation of graphite oxide) is at the expense of double bonds in the graphite. The effect of this is the degradation of the π set and the change in the mechanism of conductivity of the current in the obtained graphite oxide (from electron to semiconductive). A further reduction results in a partial regeneration of the structure of the π bonds by removing the oxygen from the structure and a return to electron conductivity which is decreasing with an increase in the temperature in the obtained reduced graphene oxide.

### 3.3. Resistance in Varying Gaseous Atmospheres (Hydrogen, Nitrogen Dioxide and Carbon Dioxide in a Carrier Gas)

#### 3.3.1. Detection of Hydrogen

**Figure 13:**
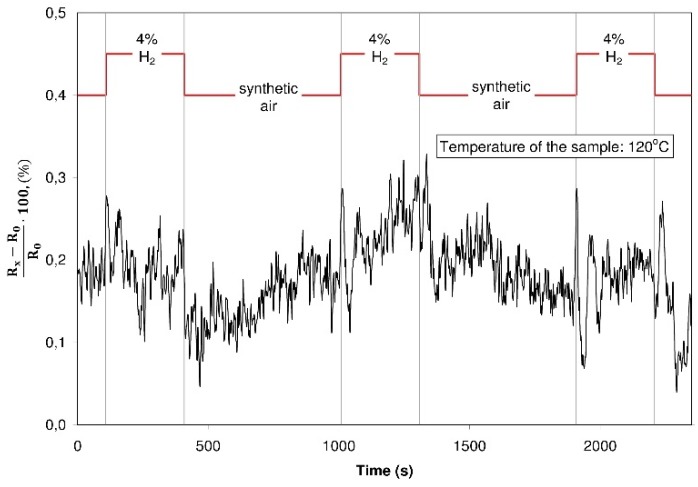
The resistance of the structure with graphite oxide during dosing of a different gaseous atmosphere (hydrogen 4% in a synthetic air/synthetic air).

**Figure 14:**
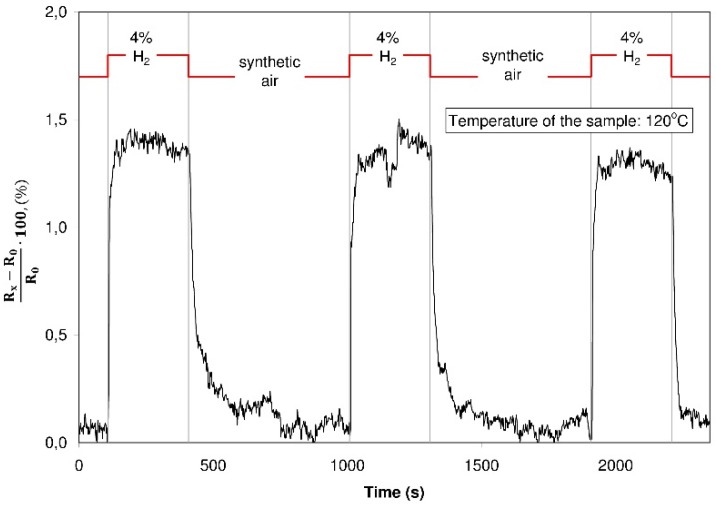
The resistance of the structure with graphene oxide during dosing of a varying gaseous atmosphere (hydrogen 4% in a synthetic air/synthetic air).

Subsequent experiments were devoted to investigations concerning the sensor properties of the structures with graphite oxide and graphene oxide in varying gaseous atmospheres. As the first test, the resistance was recorded during an alternate dosing of hydrogen with a concentration of 4% in synthetic air. These investigations were executed at various temperatures within the 30 °C to 150 °C range. The resistance of the graphite oxide structure did not change in spite of changes in the composition of the surrounding atmosphere (an exemplary characteristic plot is shown in Figure 13) whereas the resistance of the structure with graphene oxide increased distinctly during the hydrogen dosing (Figure 14).

The dynamic change of the resistance is in this case considerable, the time of reaction of the structure to its contact with the hydrogen is evidently shorter than its reaction to the contact with synthetic air (the time of detoxication). Such a character of the changes may be explained by the formation of dangling bonds in the reduction process. Favorable spots for the adsorption of the gases are created in the structure contributing to electric response changes; hydrogen adsorbing onto the structure transfers electrons and as a consequence the resistance of the structure is increasing [40]. This fast adsorption of hydrogen from the structure proves that the weak van der Waals interactions take place and that the hydrogen undergoes a physical sorption.

**Figure 15:**
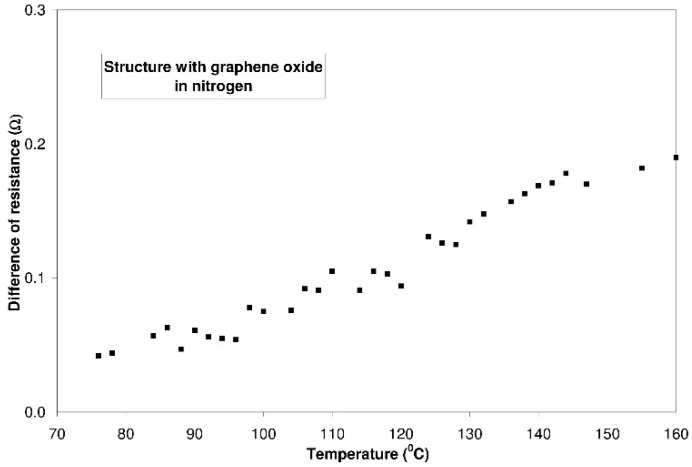
Differences (between resistance during dosing 4% in nitrogen and resistance during dosing 0% hydrogen in nitrogen) in the resistance of the structure with graphite oxide as a function of temperature.

**Figure 16:**
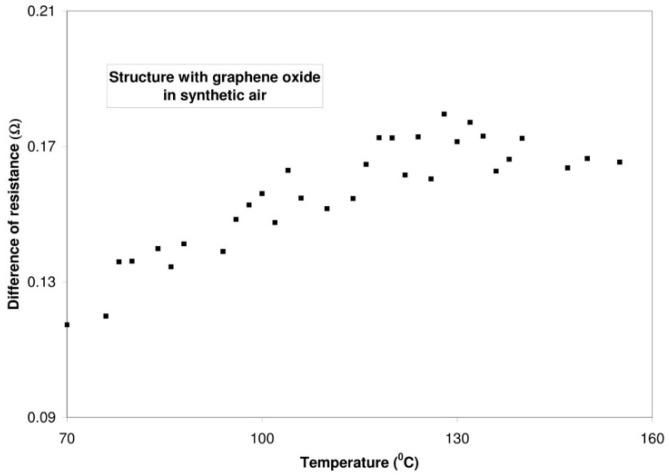
Differences (between resistance during dosing 4% in synthetic air and resistance during dosing 0% hydrogen in synthetic air) in the resistance of the structure with graphite oxide as a function of temperature.

The difference between the resistance during the dosing of nitrogen or synthetic air and the resistance during the dosing of hydrogen (4% in a carrier gas) increases (Figure 15 and Figure 16). It is caused by the generation of the surface defect (inside a given particle) due to the rising temperature. The increasing appropriate surface of nanomaterials leads to an increase in the amount of unsaturated coordinating spots which can cooperate with hydrogen.

#### 3.3.2. Detection of Nitrogen Dioxide

**Figure 17:**
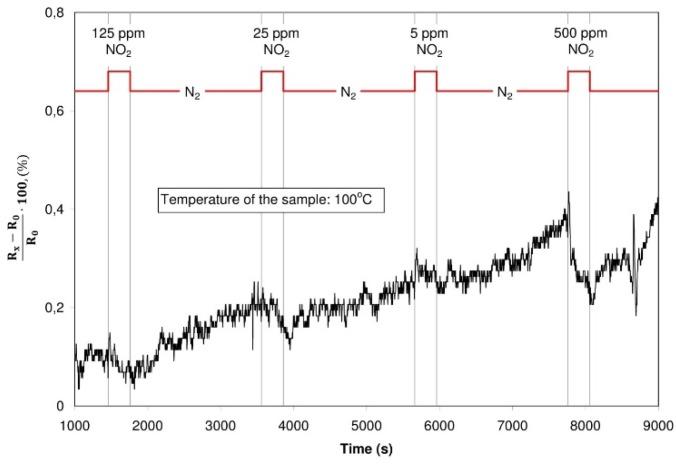
Changes of the resistance of the structure with graphite oxide (dosed atmosphere: nitrogen dioxide with concentration of 125 ppm, 25 ppm, 5 ppm and 500 ppm, temperature of the sample: 100 °C)

**Figure 18:**
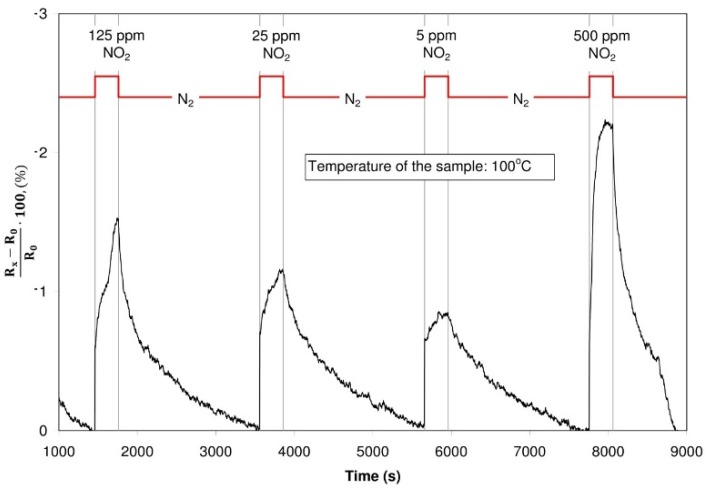
Changes of the resistance of the structure with graphene oxide (dosed atmosphere: nitrogen dioxide with concentration of 125 ppm, 25 ppm, 5 ppm and 500 ppm, temperature of the sample: 100 °C).

The sensor structure with graphene oxide was characterized by changes of the resistance during the dosing of nitrogen oxide. In such a case, both during alterdosing nitrogen oxide with various concentrations in the nitrogen, and nitrogen oxide in synthetic air, the resistance of the structure dropped in the case of dosing nitrogen dioxide. The structure with graphite oxide did not change its resistance in spite of changes of dosing gases. Exemplary characteristics are presented in Figure 17 and Figure 18.

In contact with the structure, nitrogen dioxide behaves like a donor of electrons, reducing the resistance of the structure [20,21,41]. The influence of nitrogen dioxide ought to be treated as a process of physical sorption, in which the conductivity of the sensor is increased due to electrostatic effect.

#### 3.3.3. Detection of Carbon Dioxide

**Figure 19:**
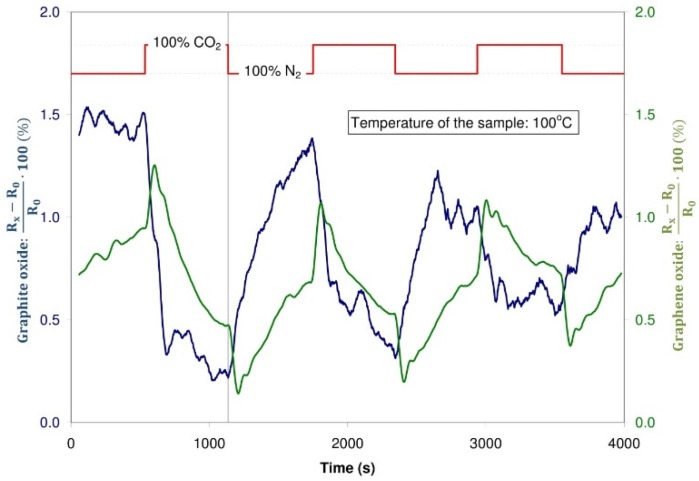
Changes of the resistance of the sensor structure with graphite oxide and graphene oxide during dosing CO_2_ and N_2_, temperature of the sample: 100 °C.

During the experiments, neither the structure with graphite oxide nor the structure with graphene oxide changed their resistance due to their contact with carbon dioxide in a distinct way. Insignificant changes of resistance were recorded (Figure 19), but most probably, these changes were due to a difference in the thermal conductivity of CO_2_ and the carrier gas: nitrogen and synthetic air.

## 4. Conclusions

The paper deals with a technique for obtaining graphite oxide by applying Hummer’s method and the technique of getting (on this graphite oxide) reduced graphene oxide. The paper presents the results of investigations concerning the chemical and physical properties of the obtained materials.

The investigations performed by means of the SEM and AFM techniques have proved that the surfaces of graphite oxide were evidently more developed than the surfaces of reduced graphene oxide. The thermal reduction involved a separation of numerous functional groups from graphite oxide, resulting in a change of the chemical composition of the obtained material. These changes were confirmed by three methods of measurements, independent of each other, *viz.* XPS, EDX and AE methods, which confirmed a considerable reduction in the amount of oxygen in the graphene oxide. Changes caused by the reduction were also confirmed by Raman’s Spectrometry and the methods TG/DTG as well as DSC.

Investigations on the sensitivity of the electric properties of the resistance structures based on graphite oxide and reduced graphene oxide to chosen gases were also performed. Generally, it may be assumed that the possibility of using graphite oxide for gas sensors is rather limited. It seems that there are considerable perspectives for applying reduced graphene oxide for detecting NO_2_ in atmospheres of synthetic air (and in the atmospheres of nitrogen, too). The investigations have proved that a resistance structure with a layer of reduced graphene oxide can react to NO_2_ already at a concentration of ppb in an atmosphere of carrier gas. This provides options for its practical use in sensing of nitrogen dioxide obtained by means of the method suggested in this paper.
